# Using Serum Metabolomic Signatures to Investigate Effects of Acupuncture on Pain-Fatigue-Sleep Disturbance in Breast Cancer Survivors

**DOI:** 10.3390/metabo14120698

**Published:** 2024-12-10

**Authors:** Hongjin Li, Ardith Z. Doorenbos, Yinglin Xia, Jun Sun, Hannah Choi, Richard E. Harris, Shuang Gao, Katy Sullivan, Judith M. Schlaeger

**Affiliations:** 1 College of Nursing, University of Illinois Chicago, 845 S. Damen Avenue, Chicago, IL 60612, USA; ardith@uic.edu (A.Z.D.); hchoi213@uic.edu (H.C.); ksulli33@uic.edu (K.S.); jschlaeg@uic.edu (J.M.S.); 2 University of Illinois Cancer Center, 818 South Wolcott Ave, Chicago, IL 60612, USA; junsun7@uic.edu; 3 College of Medicine, University of Illinois Chicago, 1853 W Polk St, Chicago, IL 60612, USA; yxia@uic.edu (Y.X.); sgao20@uic.edu (S.G.); 4 Susan Samueli Integrative Health Institute, School of Medicine, University of California at Irvine, 856 Health Sciences Rd Suite 2600, Irvine, CA 92617, USA; richareh@uci.edu; 5 Department of Anesthesia and Perioperative Care, School of Medicine, University of California at Irvine, 333 City Blvd, Orange, CA 92868, USA

**Keywords:** acupuncture, breast cancer, fatigue, metabolomics, pain, psychoneurological symptom cluster, sleep disturbance

## Abstract

Background/Objectives: Acupuncture is an efficacious integrative therapy for treating pain, fatigue, and sleep disturbance (the psychoneurological symptom cluster) in breast cancer survivors. However, the mechanisms underlying its effects remain unclear, and related metabolomics studies are limited. This study aimed to examine serum metabolite changes after acupuncture and their relationships to symptom improvement. Methods: Forty-two breast cancer survivors experiencing pain, fatigue, and sleep disturbance participated in a single-arm acupuncture trial. They received a 10-session acupuncture intervention over 5 weeks. Fasting blood samples and symptom surveys were collected before and after the acupuncture intervention, and untargeted metabolomics profiling was conducted on serum samples. Mixed-effects models adjusting for covariates (age, race, body mass index, and antidepressant use) were applied for analysis. Results: After acupuncture, there was a significant reduction in the psychoneurological symptom cluster (mean reduction = −6.2, *p* < 0.001).Bonferroni correction was applied to 40 independent metabolite clusters (α = 0.00125); cysteine-glutathione disulfide (*p* = 0.0006) significantly increased, and retinal (*p* = 0.0002) and cis-urocanate (*p* = 0.0005) were significantly decreased. Dimethyl sulfone (*p* = 0.00139) showed a trend towards reduction after acupuncture and its change (*p* = 0.04, β =1.97) was positively associated with reduction in the psychoneurological symptom cluster. Also, increased lauroylcarnitine (*p* = 0.0009) and decreased cytosine (*p* = 0.0008) can modulate the therapeutic effects of acupuncture. Conclusions: Acupuncture demonstrates beneficial effects on the psychoneurological symptom cluster in breast cancer survivors. Dimethyl sulfone may be a promising mediator in the relationship between acupuncture and psychoneurological symptoms, while acylcarnitine metabolism may modulate the therapeutic effect of acupuncture.

## 1. Introduction

Breast cancer survivors often face a wide range of ongoing health challenges during survivorship. Between 24% and 68% of breast cancer survivors report experiencing a cluster of psychoneurological symptoms during and after treatment, including pain, fatigue, and sleep disturbance [1,2,3]. These symptoms can persist for five to 10 years, significantly impacting functional status and quality of life [4,5,6]. To improve the overall well-being of breast cancer survivors, it is essential to develop and implement effective interventions to manage these persistent symptoms.

Acupuncture is a promising integrative therapy for treating cancer-related symptoms [7]. Evidence from randomized controlled trials (RCTs) supports its efficacy in reducing joint pain [8], fatigue [9], and hot flashes [10] in breast cancer survivors. A meta-analysis of 20 selected trials (with 1709 participants) indicated that acupuncture was more effective than controls in reducing pain (standardized mean difference [SMD] = −0.60) and fatigue (SMD = −0.62) in breast cancer survivors [11]. Despite promising results for acupuncture’s ability to manage isolated symptoms, few comprehensive trials have been performed to assess the simultaneous impacts of acupuncture on multiple psychoneurological symptoms [12,13,14]. Also, only a limited number of studies have thoroughly investigated the underlying mechanisms behind these effects [15]. Understanding how acupuncture influences the interconnected pathways of psychoneurological symptoms is crucial for developing more effective and targeted interventions.

Metabolomics profiling is a powerful tool that is beginning to be explored for acupuncture research and can capture hundreds of key molecules simultaneously, thus identifying diverse metabolic pathways affected by acupuncture [16]. Metabolomics profiling can also reveal metabolic changes resulting from genetic variations and the time–effect relationship between acupuncture treatment and symptom changes [17]. Several human studies have used metabolomics profiling to better understand the mechanisms involved in acupuncture at the ‘Zusanli’ acupoint (Stomach 36) [18], and in the treatment of hypertension [19] and depression [20]. Also, in one study of mice receiving chemotherapy for breast cancer [21], acupuncture was found to regulate the differential metabolites related to phenylalanine, tyrosine, and tryptophan biosynthesis, and the metabolism of taurine and hypotaurine, phenylalanine, and beta-alanine. These studies suggest that acupuncture regulates hypothalamic microinflammation [19,22] and multiple metabolic pathways [18]. However, few studies have investigated both the effect of acupuncture on metabolites and the mechanisms of acupuncture involved in the treatment of psychoneurological symptoms in breast cancer survivors [15]. Our study aims to help fill this gap by focusing on three key objectives: (1) examine changes in psychoneurological symptoms after acupuncture, (2) analyze alterations in serum metabolites after acupuncture, and (3) identify relationships between changes in metabolites and corresponding changes in psychoneurological symptoms. By pursuing these objectives, we seek to provide a deeper understanding of how acupuncture influences both the biochemical and symptomatic profiles of breast cancer survivors. Findings may enable better targeted and more effective treatments for psychoneurological symptoms in patients with breast cancer.

## 2. Materials and Methods

### 2.1. Participants and Study Design

This study was an exploratory, one-arm, phase II trial of acupuncture for the treatment of psychoneurological symptoms in breast cancer survivors. A total of 42 participants received a 5-week, 10-session acupuncture intervention. Participants were recruited from the Breast Oncology Clinic at University of Illinois Hospital & Health Sciences System (UI Health), the University of Illinois Cancer Center (UICC) Survivorship Clinic, and breast cancer support groups. The study was registered with Clinical Trials.gov (NCT05417451) and was approved by the University of Illinois Chicago Human Research Protection Office (IRB20210840).

Eligible participants met the following criteria: (1) age 18 years or older; (2) women with histologically confirmed stage 0-III ER/PR-positive, HER2-negative breast cancer; (3) currently receiving adjuvant endocrine therapy; (4) completed primary cancer treatment (e.g., surgery, radiotherapy, or chemotherapy) at least 3 months previously but within the last 5 years; (5) reported pain, fatigue, and sleep disturbance in the past month, with an average severity rating of ≥3 on a 0–10 numeric rating scale for at least two of the three symptoms; and (6) able to read and speak English. Participants were excluded if they (1) had a bleeding disorder, (2) were physically or cognitively unable to complete the study activities, or (3) were pregnant.

### 2.2. Procedures

The research team obtained a list of potentially eligible breast cancer survivors from oncologists at UI Health and the UICC Survivorship Clinic. We reviewed participants’ electronic health records (EHRs) to identify those who met the first three inclusion criteria. We then contacted potential participants via email and text messaging that contained information about the study. For those who expressed interest, the research coordinator either met them in the clinic waiting room or contacted them by telephone to introduce the study and conduct full eligibility screening. Eligibility was assessed through communication with healthcare providers, review of the EHR, and then asking potential participants to rate their levels of pain, fatigue, and sleep disturbance over the past month on a 0 to 10 scale. Once eligibility was confirmed, we scheduled a baseline study visit with each participant.

At baseline (week 0), after providing informed consent, participants completed self-report measures of demographics, clinical history, and symptoms. Prior to the first acupuncture session, a fasting blood sample (10 mL) was collected from each participant between 8:00 and 11:00 a.m. using a red-top clot activator silicone coated Vacutainer^®^ tube. The samples laid undisturbed at room temperature for 30 min to clot, and where then centrifuged at 2000× *g* for 15 min to separate the serum. The serum was then aliquoted into clean polypropylene tubes using a Pasteur pipette and stored at minus 80 °C until further processing. Then, all participants began the 5-week, 10-session acupuncture intervention within 24 h of having their blood drawn. After the 10th acupuncture treatment, self-report measures were completed and a fasting blood sample (10 mL) was drawn for the second time.

### 2.3. Acupuncture Intervention

The acupuncture intervention was designed following the Standards for Reporting Interventions in Controlled Trials in Acupuncture (STRICTA) guidelines [23]. The treatment protocol consisted of 10 sessions occurring twice per week over 5 weeks, with at least 1 day in between sessions. Participants received (1) a standardized acupuncture protocol targeting generalized pain, fatigue, sleep disturbance, depression, and anxiety and (2) additional acupuncture points to address up to three more of their most painful areas. Details of the acupuncture points and protocol can be found in our protocol article [24]. Needles were retained for 30 min and were gently rotated three times during the session (at 10, 20, and 30 min) to facilitate the movement of *qi* and blood. A single-size Korean DBC^®^ 0.25 × 40 mm stainless-steel acupuncture needle with a wound stainless-steel handle was used for all insertions. The intervention was administered by a licensed acupuncturist certified by the National Certification Commission for Acupuncture and Oriental Medicine (NCCAOM), who closely monitored each participant for needle shock and other potential side effects such as bruising or dizziness. Any adverse events were recorded in an adverse event log.

### 2.4. Metabolite Profiling and Quality Control

Paired serum samples were collected from the 42 participants at baseline and after the 10th acupuncture treatment. In total, 84 serum samples were analyzed using global untargeted metabolomics profiling by Metabolon Metabolomics Laboratory (Morrisville, NC). The sample preparation was carried out using the automated MicroLab STAR^®^ system (Hamilton Company, Salt Lake City, UT, USA). Sample extraction was divided into five fractions: two for analysis by two separate reverse phase (RP)/ultra-performance liquid chromatography (UPLC)-mass spectroscopy (MS)/MS methods with positive ion mode electrospray ionization (ESI), one for analysis by RP/UPLC-MS/MS with negative ion mode ESI, one for analysis by hydrophilic interaction liquid chromatography (HILIC)/UPLC-MS/MS with negative ion mode ESI, and one reserved as a backup. The RP columns were used to analyze non-polar to moderately polar metabolites, while the HILIC column targeted polar compounds.

All methods utilized a Waters ACQUITY UPLC and a Thermo Scientific Q-Exactive high resolution/accurate mass spectrometer interfaced with a heated electrospray ionization (HESI-II) source and an Orbitrap mass analyzer operated at 35,000 mass resolution for MS1 and 15,000 mass resolution for MS2. Raw data were extracted, peaks were identified, and quality controls were processed using Metabolon’s hardware, software, and published protocol [25]. Metabolon maintains a library based on authenticated standards that contain the retention time/index, mass-to-charge ratio, and chromatographic data (including MS/MS spectral data) for all molecules present in the library. Peaks were quantified using the area under the curve.

A total of 1465 serum metabolites were detected, including 257 biochemicals that have not been named or confirmed. We carried out further quality control for the metabolite data before analysis. Among the named metabolites, we further excluded 155 for which more than 50% of samples had values below the detection limit. A total of 1053 identified metabolites with satisfactory detection rates were entered into the analysis. Metabolites with less than 50% missing data underwent imputation and normalization using the best Normalize package in R for continuous variable analyses [26].

### 2.5. Measures

#### 2.5.1. Demographics and Clinical Characteristics

Sociodemographic characteristics such as age, race, education, income, and marital status were collected via a self-report questionnaire. Clinical status characteristics such as height, weight, disease stage, and types of treatments were also collected at baseline.

#### 2.5.2. PROMIS Measures

Patient-Reported Outcomes Measurement Information System (PROMIS)-29 (v1.0) questionnaires were used to collect participant experiences. PROMIS surveys are reliable measures of symptom experience among cancer patients, with good internal consistency and convergent validity (Cronbach alphas: 0.86–0.96) [27,28]. The PROMIS Pain Interference subscale (4 items) focuses on pain interference, defined as the interference of pain in daily activities involving physical, psychological, and social functioning [29]. The PROMIS Fatigue subscale (4 items) assesses fatigue from mild subjective feelings of tiredness to an overwhelming and sustained sense of exhaustion [30]. The PROMIS Sleep Disturbance subscale (4 items) is used to assess perceived sleep quality, sleep depth, and restoration associated with sleep; perceived difficulties and concerns with getting to sleep or staying asleep; and perceived adequacy of and satisfaction with sleep [31].

#### 2.5.3. Dietary Screening and Control

At baseline and week 6, participants completed the Dietary Screener Questionnaire (DSQ) [32], which includes detailed descriptions of the types and amounts of foods and beverages consumed. We instructed participants to maintain their overall dietary patterns (alcoholic/caffeinated beverage consumption, and dietary supplement usage) during the study.

### 2.6. Statistical Analysis

Sample sizes were calculated based on the paired Wilcoxon signed-rank test between participants pre- and post-acupuncture. Based on the median absolute deviation of differences of primary outcomes from our feasibility study, we estimated the pre–post effect size for the psychoneurological symptom cluster to be 0.9, while the pre–post effect size for pain, fatigue, and sleep disturbance ranged from 0.50 to 0.98. A 2-sided paired Wilcoxon signed-rank test for the presence of change with 35 patients would have 80% power to detect an effect size of 0.5 with an alpha of 0.05. To account for a 20% attrition rate for the acupuncture intervention, we recruited 42 participants.

The sociodemographic and clinical characteristics of the participants are presented as mean ± standard deviation (SD) for continuous variables and N (%) for categorical variables. The paired Wilcoxon signed-rank test was used to compare the psychoneurological symptom cluster score (i.e., composite score of pain, fatigue, and sleep disturbance) before and after acupuncture. The DSQ was converted to calculate the predicted intake of fiber, added sugars, sugar-sweetened beverages, dairy, fruits, and vegetables using the data processing and scoring procedures developed for the NHANES 2009-10 DSQ [32]. Missing data were imputed using median values. The predicted intake among participants is presented as mean ± SD.

To identify significant changes in metabolites before and after acupuncture, we used mixed-effects linear regression to assess the differential profiling of metabolites while accounting for repeated measures and controlling for age, race, body mass index (BMI), and antidepressant use as confounding variables. We further investigated whether changes in these metabolites were associated with changes in the psychoneurological symptom cluster after acupuncture using a linear regression model controlled for the same variables.

To identify significant moderators, we used linear mixed modeling with interaction terms. This approach enabled us to test for significant interaction effects, specifically examining whether certain metabolites moderated and influenced the impact of acupuncture on psychoneurological symptoms without being directly altered by the acupuncture treatment itself. Since metabolite levels naturally fluctuated over the course of the treatment period (independent of the acupuncture intervention), these analyses helped to clarify how changing metabolite levels during the treatment contributed to the observed effects of acupuncture on psychoneurological symptoms. We developed a model for the overall participants, as follows:

Model: Psychoneurological symptom cluster = metabolites + time + metabolites ∗ time + age + race + BMI + antidepressants

To correct for multiple comparisons, we conducted a principal component analysis (PCA) on all metabolites to determine the number of independent metabolite clusters. All *p*-values were then adjusted for the number of independent clusters using a Bonferroni correction. The PCA identified 40 principal components, which explained 99.5% of the total variance. Thus, metabolites with a *p*-value < 0.00125 (0.05/40) were considered statistically significant. All analyses were conducted using R.

## 3. Results

### 3.1. Sociodemographic and Clinical Characteristics of Participants

Table 1 summarizes the sample demographics and clinical characteristics at baseline. Participants were women aged 52.0 ± 10.5 years, with an average BMI of 30.0 ± 6.0. The study population was notably diverse, with 50.0% identifying as White, 40.5% as Black/African American, and 21.4% as Hispanic. Most participants had attained at least a bachelor’s degree (64.3%). Fifteen (35.7%) were married and 22 (52.4%) had a household income of less than USD 55,000. Seventeen participants were diagnosed with early-stage breast cancer, with 40.5% having Stage 1 and 40.5% having Stage 2. Regarding types of treatment received, all the participants were taking endocrine therapy and 64.3% previously had chemotherapy. Ten participants (23.8%) reported antidepressant use.

Regarding dietary intake, at week 6, participants’ predicted intake of fiber, sugar-sweetened beverages, dairy products, fruits, and vegetables remained the same as baseline, while intake of total added sugars had a minor increase to 12.0 g/day (see Table 2).

### 3.2. Symptoms Before and After Acupuncture

Most participants (93.5%) completed all 10 acupuncture sessions. Pain interference, fatigue, and sleep disturbance scores at baseline and post-intervention are displayed in Table 3. Results from the Wilcoxon signed-rank test revealed significant reductions in PROMIS T scores following the 10-session acupuncture intervention. Participants experienced substantial decrease in pain interference (mean reduction = −5.2, *p* < 0.001), fatigue (mean reduction = −8.0, *p* < 0.001), sleep disturbance (mean reduction = −7.2, *p* < 0.001), and the psychoneurological symptom cluster (mean reduction = −6.2, *p* < 0.001).

### 3.3. Changes in Metabolites in 6 Weeks After the 10-Session Acupuncture Treatment

**Figure 1:**
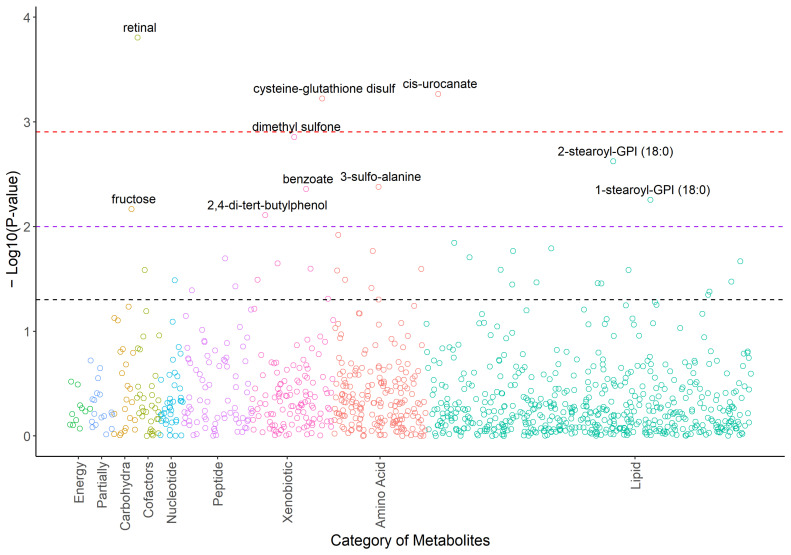
Plot of −log10Ps for the metabolomic profiles in response to the acupuncture treatments. Note. Ps were calculated based on the mixed effects models adjusted for age, race, BMI, and use of antidepressants as confounding variables. Associations with raw *p* < 0.05 are labeled with chemical names. Black dashed line: raw *p* = 0.05; purple dashed line: raw *p* = 0.01; red dashed line: raw *p* = 0.00125.

**Figure 2:**
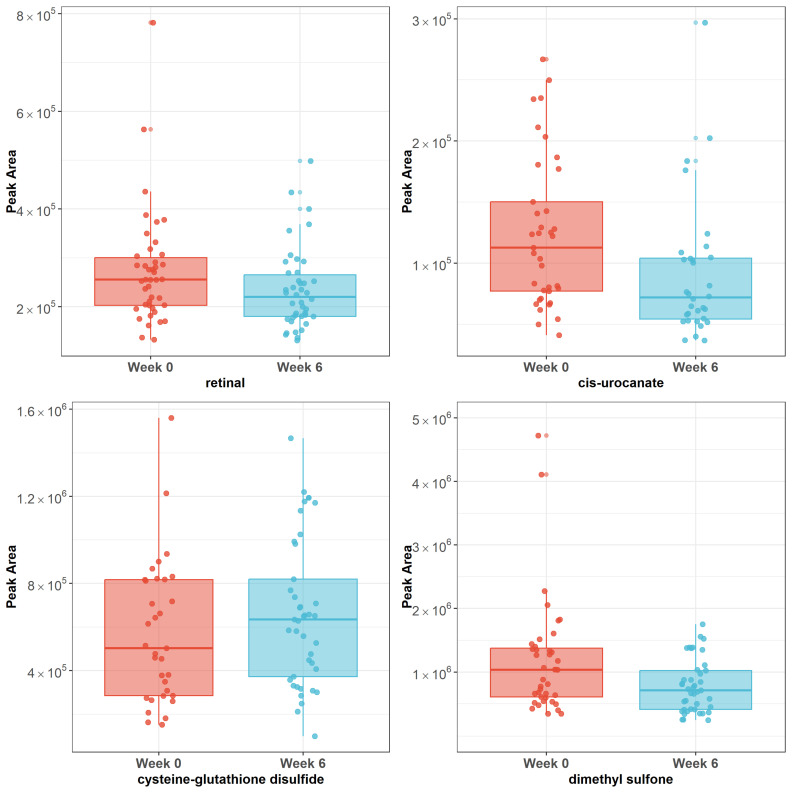
**Plots of raw peak areas of four metabolites at week 0 and week 6.** Note: Box plots represent the median ± interquartile range. Cysteine-glutathione disulfide was upregulated after completion of acupuncture (*p* = 0.0006), while retinal (*p* = 0.0002), cis-urocanate (*p* = 0.0005), and dimethyl sulfone (*p* = 0.00139) were downregulated after completion of acupuncture.

There were 40 metabolites that responded to acupuncture with raw *p* < 0.05 (Figure 1, Supplementary Table S1). The top three metabolites (retinal, cis-urocanate, cysteine-glutathione disulfide) remained significant after Bonferroni correction using a significant threshold *p* < 0.00125. Dimethyl sulfone showed a significant trend (*p* = 0.00139). Cysteine-glutathione disulfide was upregulated after the completion of acupuncture (*p* = 0.0006, β = 0.56), while retinal (*p* = 0.0002, β = −0.44), cis-urocanate (*p* = 0.0005, β = −0.65), and dimethyl sulfone (*p* = 0.00139, β = −0.56) were downregulated after the completion of acupuncture (see Figure 2).

### 3.4. Association of Changes in Serum Metabolites with Change in the Psychoneurological Symptom Cluster After Acupuncture Treatment

Among 40 metabolites that responded to acupuncture with raw *p* < 0.05, changes of dimethyl sulfone from pre-acupuncture to post-acupuncture were positively (*p* = 0.04, β = 1.97) associated with reduction in the psychoneurological symptom cluster (see Table 4). Notably, after completion of acupuncture, dimethyl sulfone was downregulated. This reduction in dimethyl sulfone was associated with improved outcomes in the psychoneurological symptom cluster.

### 3.5. Potential Metabolites That Modulate Acupuncture’s Impact on Psychoneurological Symptoms over Time

Mixed-effect analyses identified two novel metabolites with a significant metabolite-by-time interaction after Bonferroni correction (Table 5): cytosine (*p* < 0.00125, β = 3.85) and lauroylcarnitine (*p* < 0.00125, β = −3.70). The significant metabolite-by-time interactions imply that cytosine and lauroylcarnitine serve as dynamic moderators in the relationship between acupuncture treatment and psychoneurological symptoms. They suggest that breast cancer survivors with lower cytosine levels and higher lauroylcarnitine levels during the acupuncture treatment are associated with more improvements of the psychoneurological symptom cluster. We listed the top 10 metabolites in Table 5 and all the results for all metabolites in Supplementary Table S2.

## 4. Discussion

Our study demonstrates that acupuncture can alter serum metabolite levels in breast cancer survivors experiencing the psychoneurological symptom cluster of pain, fatigue, and sleep disturbance. Acupuncture was found to upregulate cysteine-glutathione disulfide. Levels of retinal, cis-urocanate, and dimethyl sulfone were reduced in serum following the 10-session acupuncture protocol. Specifically, reduction in dimethyl sulfone in serum was associated with improved outcomes of the psychoneurological symptom cluster. Cytosine and lauroylcarnitine changes during the course of acupuncture treatment play a role in moderating the treatment effect of acupuncture on psychoneurological symptoms. These findings offer insights into acupuncture’s therapeutic mechanisms involved in improving symptom management in breast cancer survivors.

Most studies have focused on examining metabolite changes before and after acupuncture. Very few have taken the crucial step of linking these metabolic changes to the underlying mechanisms by which acupuncture alleviates symptoms. Our study advances this understanding by demonstrating that dimethyl sulfone is a promising mediator of acupuncture’s effect on the psychoneurological symptoms in breast cancer survivors undergoing endocrine therapy. Dimethyl sulfone, also known as methylsulfonylmethane (MSM), is an organosulfur compound naturally present in plants, animals, and humans [33]. MSM is widely recognized for its anti-inflammatory properties and is commonly used as a dietary supplement to support joint health, connective tissue integrity, and pain relief [34]. Additionally, MSM is known to have antioxidative effects, potentially reducing oxidative stress and modulating immune function, which may contribute to its therapeutic benefits in various chronic conditions [34]. In our study, we observed a reduction in MSM levels following acupuncture treatment, and this reduction was associated with greater improvements in the psychoneurological symptom cluster. This finding suggests that MSM is worth exploring in more depth as a mediator, especially through a formal mediation analysis, to confirm whether MSM mediates the effect of acupuncture on the psychoneurological symptom cluster. It is possible that decreased MSM in serum could reflect the utilization of MSM to counteract inflammation and oxidative stress during acupuncture treatment. It is also possible that acupuncture influences the utilization of MSM in joints and/or other tissues, potentially enhancing its uptake or metabolism in areas of inflammation or pain. Further research is needed to clarify the exact mechanisms by which reduction in MSM influences psychoneurological symptom improvement in breast cancer survivors after completion of acupuncture.

Additionally, fatty lipids associated with acylcarnitine metabolism play a pivotal role in modulating the therapeutic effects of acupuncture on psychoneurological symptoms in breast cancer survivors. Specifically, increased levels of lauroylcarnitine (C12) were correlated with a reduction in psychoneurological symptoms following acupuncture. Acylcarnitines are fatty acid metabolites that play a critical role in cellular energy metabolism by facilitating the transport of long-chain fatty acids into mitochondria, where they undergo β-oxidation to produce ATP [35]. Additionally, acylcarnitines can induce mitochondrial oxidative stress, suppress the antioxidant gene HMOX1, and increase the expression of CXCL8 and the release of IL-8 [36]. The role of acylcarnitines in energy production and oxidative stress protection highlights their significance in maintaining cellular health and influencing various pathological conditions such as fatigue [37], fibromyalgia [38], and cardiovascular disease [39]. A systematic review of 276 studies indicated that patients with fatigue often have lower serum levels of acylcarnitine [37]. Furthermore, long-term administration of acylcarnitine has been shown to alleviate symptoms of chronic fatigue [37]. Our findings suggest that increasing lauroylcarnitine levels can enhance the effectiveness of acupuncture in reducing psychoneurological symptoms. This indicates that acylcarnitine metabolism may be a key target for optimizing acupuncture treatment. L-acetylcarnitine supplementation or monitoring acetylcarnitine levels during acupuncture may potentially improve clinical outcomes. Understanding the related metabolic pathways involved in acupuncture will help us identify biomarkers that predict treatment response, allowing for more personalized acupuncture interventions. Future research should focus on how lauroylcarnitine affects the efficacy of acupuncture on psychoneurological symptoms and explore the potential for integrating metabolic profiling into clinical practice to maximize the benefits of acupuncture.

Retinal, also known as retinaldehyde, is one form of vitamin A. It plays a crucial role in vision and the visual cycle by acting as a chromophore in photoreceptor cells, where it undergoes isomerization in response to light, enabling the process of vision [40]. However, exposure to excess retinol, the precursor to retinal, can lead to an overproduction of all-trans-retinal. This excessive accumulation can be harmful, as all-trans-retinal is a reactive aldehyde that can form toxic adducts with cellular proteins and lipids. These toxic byproducts can disrupt cellular function, leading to oxidative stress, inflammation, and ultimately degeneration of retinal cells. Similarly, Xia et al. observed that retinol levels were downregulated following acupuncture in women undergoing in vitro fertilization (IVF) [41]. Both our findings and Xia et al.’s emerging evidence highlight the importance of understanding the intricate balance of vitamin A derivatives like retinal and retinol in various physiological processes beyond vision, including reproduction, cellular health, as well as psychoneurological symptoms [41]. These findings also underscore the need for further research in how acupuncture might influence vitamin A metabolism and contribute to overall health outcomes.

Cysteine-glutathione disulfide (CySSG) is a glutathione (GSH) derivative, comprised of the oxidized form of free glutathione tripeptide linked via a disulfide bond to L-cysteine. It is produced by the reaction of oxidized glutathione (GSSG) and cysteine [42]. GSH is a powerful antioxidant and plays a critical role in the body’s defense against oxidative stress. Changes in GSH and CySSG concentrations are correlated with age and health [43]. Recent systematic reviews showed that acupuncture regulates oxidative stress by lowering the lipid peroxidation and activating the antioxidant enzyme system [44,45]. Higher glutathione peroxidase activity, increased GSH, and increased GSH/GSSG were found in studies of acupuncture for the treatment of obesity. Increased GSH levels were also observed in our previous study using acupuncture to treat psychoneurological symptoms [46], as well as in a study using electroacupuncture for depression [20]; CySSG was upregulated after the acupuncture intervention. However, in our current study, both GSH and GSSG were not measured in the untargeted panel list. Therefore, we could only use the glutathione derivative CySSG to reflect changes in the glutathione pathway. Future research is needed to investigate the potential role of acupuncture in managing oxidative stress and enhancing the body’s antioxidant capacity, particularly through the glutathione pathway.

Cis-urocanate is an intermediate of the histidine degradation pathway. It is synthesized from histidine by uncoupling ammonia. Further degradation of cis-urocanate leads to production of glutamate. Cis-urocanate has a protective function for the skin and may play a role in UV-induced immunosuppression by generating reactive oxygen species [47]. Cis-urocanate also suppresses cell-mediated immunity and contributes to a systemic anti-inflammatory effect [48]. Histidine is an essential amino acid shown to enhance insulin sensitivity, reduce body fat and waist circumference, and decrease oxidative stress and systemic pro-inflammatory markers [49]. Despite the important role that cis-urocanate played in these processes, no studies have reported changes in cis-urocanate levels after acupuncture compared to baseline. Of note, one animal study found that L-histidine was reduced after acupuncture [50]. The specific impact of acupuncture on cis-urocanate levels and histidine metabolism remains unexplored. Further studies are needed to investigate how acupuncture may influence the histidine degradation pathway, including the role of cis-urocanate.

The strengths of this study include our consideration of age, race, BMI, and antidepressant status in the mixed-effects model, as well as our assessment of dietary patterns. However, our study has several limitations. First, while we accounted for important demographic and clinical variables, other potential confounding factors, such as postmenopausal status, variations in lifestyle, differences in physical activity, and sleep status were not controlled in the analysis. Since these factors can significantly influence metabolite levels, it is critical for future studies to control for these variables in the study design. Second, the lack of a control group may affect the robustness of our conclusions regarding the specific impact of acupuncture on psychoneurological symptoms. The main purpose of this study is to find signals for significant metabolites changes related to the psychoneurological symptom cluster pre- and post-acupuncture. These signals and biomarkers will then inform a larger randomized phase III trial, which will include a sham acupuncture control group. Lastly, the timing of the blood sample collection post-acupuncture varied widely, ranging from 24 h to 3 days. Samples collected within 24 h may capture the acute effects of acupuncture, while those collected between 24 h to 3 days may reflect more sustained, chronic effects. This variability in timing could impact the interpretation of how acupuncture influences metabolic changes and psychoneurological symptoms over different time frames. Standardizing the timing of blood sample collection in future studies could help delineate the acute versus chronic effects of acupuncture and provide a clearer understanding of its impact on metabolite profiles and symptom relief.

## 5. Conclusions

Acupuncture demonstrates a significant capacity to alleviate pain, fatigue, and sleep disturbances experienced by breast cancer survivors undergoing endocrine therapy. Our global untargeted metabolomic profiling study revealed that acupuncture increased serum levels of cysteine-glutathione disulfide while reducing levels of retinal, cis-urocanate, and MSM. Especially, changes in MSM levels are associated with both acupuncture treatment and changes in psychoneurological symptom cluster. Our findings underscore the crucial role of MSM and acetylcarnitine metabolism, particularly lauroylcarnitine, in the therapeutic mechanisms underlying the effects of acupuncture on the symptom cluster of pain, fatigue, and sleep disturbance. Further mediation analysis is needed to confirm the role of MSM as a mediator in managing psychoneurological symptoms in breast cancer survivors undergoing endocrine therapy.

## Figures and Tables

**Table 1:** Baseline characteristics of the participants (*N* = 42).

Sociodemographic Characteristics	Mean ± SD or N (%)
**Sex**	
Female	42 (100%)
**Age**	52.0 ± 10.5
**BMI**	30.0 ± 6.0
**Ethnicity**	
Hispanic	9 (21.4%)
Non-Hispanic	33 (78.6%)
**Race**	
Asian	1 (2.4%)
Black/African American	17 (40.5%)
White/Caucasian	21 (50.0%)
Other	3 (7.1%)
**Education level**	
Less Than High School	1 (2.4%)
High School Graduate	5 (11.9%)
Some College	7 (16.7%)
Bachelor’s Degree	14 (33.3%)
Some Graduate School	1 (2.4%)
Graduate School	14 (33.3%)
**Marital status**	
Never Married	14 (33.3%)
Married/Living with Partner	15 (35.7%)
Divorced/Separated	11 (26.2%)
Widowed	2 (4.8%)
**Household income**	
Less than USD 35,000	12 (28.6%)
USD 35,000–USD 55,000	10 (23.8%)
USD 55,000–USD 100,000	12 (28.6%)
More than USD 100,000	8 (19.0%)
**Stage of cancer**	
0	2 (4.8%)
1	17 (40.5%)
2	17 (40.5%)
3	6 (14.3%)
**Types of treatments**	
Endocrine therapy	42 (100%)
Radiation therapy	33 (78.6%)
Chemotherapy	27 (64.3%)
**Antidepressant use**	
Yes	10 (23.8%)
No	22 (52.4%)
Unknown	10 (23.8%)

Note. BMI = body mass index; N = number; SD = standard deviation.

**Table 2:** Baseline and week 6 dietary report (*N* = 42).

Mean ± SD	Week 0 (Baseline)	Week 6
Predicted intake of fiber (gm) per day *	13.3 ± 1.2	13.3 ± 1.5
Predicted intake of total added sugars (tsp equivalents) per day *	11.7 ± 1.6	12.0 ± 2.5
Predicted intake of added sugars from sugar-sweetened beverages (tsp equivalents) per day *	4.1 ± 0.9	4.1 ± 0.6
Predicted intake of dairy (cup equivalents) per day *	1.3 ± 0.3	1.3 ± 0.5
Predicted intake of fruits and vegetables including legumes and excluding French fries (cup equivalents) per day *	1.9 ± 0.4	1.9 ± 0.3
Predicted intake of vegetables including legumes and excluding French fries (cup equivalents) per day *	1.4 ± 0.3	1.4 ± 0.4
Predicted intake of fruits (cup equivalents) per day *	0.5 ± 0.1	0.5 ± 0.1
Frequency of red meat intake per day	0.2 ± 0.3	0.3 ± 0.4
Frequency of processed meat intake per day	0.2 ± 0.2	0.2 ± 0.3

Note. N = number; SD = standard deviation. * Predicted intake values are calculated using scoring algorithms developed by Thompson et al. (2017) [32].

**Table 3:** PROMIS outcomes before and after acupuncture treatment (N = 42).

Outcomes	Before (T Score ± SD)	After (T Score ± SD)	Change * (T Score ± SD)	Change ** (T Score ± SD)
Pain interference	58.8 ± 9.0	53.6 ± 9.2	5.2 ± 6.4 (*p* < 0.001)	6.0 ± 6.4 (*p* < 0.001)
Fatigue	56.7 ± 8.6	48.8 ± 8.6	8.0 ± 8.7 (*p* < 0.001)	9.3 ± 8.8 (*p* < 0.001)
Sleep disturbance	58.7 ± 6.3	51.5 ± 8.0	7.2 ± 9.3 (*p* < 0.001)	6.6 ± 9.4 (*p* < 0.001)
Psychoneurological symptom cluster (pain interference + fatigue + sleep disturbance)	58.1 ± 6.3	51.3 ± 6.5	6.8 ± 6.6 (*p* < 0.001)	6.2 ± 6.7 (*p* < 0.001)

Note. N = number, SD = standard deviation. * Student’s *t*-test; ** Wilcoxon signed-rank test.

**Table 4:** Association between changes in metabolites with changes of psychoneurological symptoms after completion of acupuncture.

Rank	Metabolites	Sub-Pathway	β *	*p* Value
**1**	dimethyl sulfone	Chemical	1.97	0.047
**2**	retinal	Vitamin A Metabolism	2.27	0.136
**3**	cysteine-glutathione disulfide	Glutathione Metabolism	−0.29	0.796
**4**	cis-urocanate	Histidine Metabolism	−0.21	0.825

Note. * β is the regression coefficient of the intervention in the mixed-effects model adjusted for age and race.

**Table 5:** Top 10 novel metabolites that modulate acupuncture’s impact on the psychoneurological symptom cluster over time.

Rank	Metabolites	Sub-Pathway	β *	*p* Value
**1**	cytosine	Pyrimidine Metabolism	3.85	<0.001
**2**	lauroylcarnitine (C12)	Fatty Acid Metabolism (Acyl Carnitine, Medium Chain)	−3.70	<0.001
**3**	N-acetylglycine	Glycine, Serine, and Threonine Metabolism	−3.39	0.001
**4**	5-dodecenoylcarnitine (C12:1)	Fatty Acid Metabolism (Acyl Carnitine, Monounsaturated)	−3.31	0.002
**5**	hexanoylcarnitine (C6)	Fatty Acid Metabolism (Acyl Carnitine, Medium Chain)	−3.37	0.004
**6**	2-oxoarginine	Urea Cycle; Arginine and Proline Metabolism	3.37	0.004
**7**	2-aminophenol sulfate	Food Component/Plant	3.52	0.005
**8**	pipecolate	Lysine Metabolism	3.11	0.005
**9**	acetyl carnitine (C2)	Fatty Acid Metabolism (Acyl Carnitine, Short Chain)	−3.26	0.006
**10**	2-acrylamidoglycolic acid	Chemical	3.15	0.008

Note. * β is the regression coefficient of intervention in the mixed-effects model adjusted for age, race, BMI, and use of antidepressants.

## Data Availability

The data that support the findings of this study are openly available in the manuscript and Supplementary Materials.

## Supplementary Materials

The following supporting information can be downloaded at: https://www.mdpi.com/article/10.3390/metabo14120698/s1, Supplementary Table S1: Significant Metabolite Changes After Acupuncture; Supplementary Table S2: Significant Metabolites Associated with Changes of Psychoneurological Symptoms After Acupuncture.
