# Supplementary material for: Using Serum Metabolomic Signatures to Investigate Effects of Acupuncture on Pain-Fatigue-Sleep Disturbance in Breast Cancer Survivors
Source: Metabolites. 2024 Dec 10;14(12):698. doi: 10.3390/metabo14120698 (PMC11679957; doi:10.3390/metabo14120698)
Supplement: Supplementary file 1 [file metabolites-14-00698-s001.zip › metabolites-3331281-supplementary.pdf]

Supplementary Materials

Table S1. Significant Metabolite Changes After Acupuncture

Metabolites	Sub Pathway	β*	Raw *p* value
**retinal**	Vitamin A Metabolism	-0.44	0.0002
**cis-urocanate**	Histidine Metabolism	-0.65	0.0005
**cysteine-glutathione disulf**	Glutathione Metabolism	0.56	0.0006
**dimethyl sulfone**	Chemical	-0.56	0.0014
**2-stearoyl-GPI (18:0)**	Lysophospholipid	0.64	0.0024
**3-sulfo-alanine**	Methionine, Cysteine	-0.53	0.0044
**benzoate**	Benzoate Metabolism	-0.59	0.0044
**1-stearoyl-GPI (18:0)**	Lysophospholipid	0.56	0.0055
**fructose**	Fructose, Mannose and Galactose Metabolism	0.32	0.0068
**2,4-di-tert-butylphenol**	Chemical	0.56	0.0078
**isovalerate (i5:0)**	Leucine, Isoleucine and Valine Metabolism	-0.41	0.0120
**1-palmitoyl-2-(hydroxylinoleoyl)-GPC (16:0/18:2(OH))**	Phosphatidylcholine (PC)	-0.28	0.0143
**2-palmitoleoyl-GPC (16:1)**	Lysophospholipid	0.40	0.0161
**cysteine s-sulfate**	Methionine, Cysteine, SAM and Taurine Metabolism	-0.30	0.0171
**dehydroepiandrosterone sulfate (DHEA-S)**	Androgenic Steroids	0.15	0.0171
**1-linoleoyl-GPI (18:2)**	Lysophospholipid	0.38	0.0196
**N,N-dimethyl-pro-pro**	Modified Peptides	0.30	0.0201
**1-palmitoyl-GPI (16:0)**	Lysophospholipid	0.40	0.0214
**propyl 4-hydroxybenzoate sulfate**	Benzoate Metabolism	0.35	0.0224
**ethyl beta-glucopyranoside**	Food Component/Plant	0.34	0.0252
**cystine**	Methionine, Cysteine, SAM and Taurine Metabolism	-0.34	0.0254

**1-arachidonoyl-GPI (20:4)**	Lysophospholipid	0.41	0.0259
**heptanoate (7:0)**	Medium Chain Fatty Acid	-0.47	0.0259
**retinol (vitamin A)**	Vitamin A Metabolism	0.20	0.0260
**3-hydroxyisobutyrate**	Leucine, Isoleucine and Valine Metabolism	0.35	0.0262
**ethylparaben sulfate**	Chemical	0.26	0.0321
**tiglylcarnitine (C5:1-DC)**	Leucine, Isoleucine and Valine Metabolism	-0.30	0.0322
**orotate**	Pyrimidine Metabolism, Orotate containing	0.21	0.0326
**glutarate (C5-DC)**	Fatty Acid, Dicarboxylate	0.21	0.0335
**androstenediol (3beta,17beta) monosulfate (2)**	Androgenic Steroids	0.12	0.0342
**palmitoyl dihydrosphingomyelin (d18:0/16:0)**	Dihydrosphingomyelins	-0.22	0.0347
**androstenediol (3beta,17beta) monosulfate (1)**	Androgenic Steroids	0.19	0.0348
**2-arachidonoyl-GPC (20:4)**	Lysophospholipid	0.34	0.0357
**isoleucylthreonine**	Dipeptide	0.28	0.0372
**N-acetylmethionine sulfoxide**	Methionine, Cysteine, SAM and Taurine Metabolism	0.37	0.0385
**isoleucylglycine**	Dipeptide	0.39	0.0406
**1-nervonoyl-GPC (24:1n9)**	Lysophospholipid	0.24	0.0417
**1-palmitoyl-2-linoleoyl-GPC (16:0/18:2)**	Phosphatidylcholine (PC)	0.23	0.0448
**eugenol sulfate**	Food Component/Plant	0.29	0.0489
**trans-urocanate**	Histidine Metabolism	-0.30	0.0496

Note. *β is the regression coefficient of intervention in the mixed-effects model adjusted for age, race, BMI and use of antidepressant.

Table S2. Significant Metabolites Associated with Changes of Psychoneurological Symptoms After Acupuncture

**Metabolites**	**Pathway**	**β***	**Raw *p* value**
**cytosine**	Pyrimidine Metabolism, Cytidine containing	3.86	0.0008
**laurylcarnitine (C12)**	Fatty Acid Metabolism (Acyl Carnitine, Medium Chain)	-3.70	0.0009
**N-acetylglycine**	Glycine, Serine and Threonine Metabolism	-3.39	0.0013

**5-dodecenoylcarnitine (C12:1)**	Fatty Acid Metabolism (Acyl Carnitine, Monounsaturated)	-3.31	0.0022
**hexanoylcarnitine (C6)**	Fatty Acid Metabolism (Acyl Carnitine, Medium Chain)	-3.37	0.0040
**2-oxoarginine**	Urea cycle; Arginine and Proline Metabolism	3.37	0.0042
**2-aminophenol sulfate**	Food Component/Plant	3.52	0.0047
**pipecolate**	Lysine Metabolism	3.11	0.0054
**acetylcarnitine (C2)**	Fatty Acid Metabolism (Acyl Carnitine, Short Chain)	-3.26	0.0060
**2-acrylamidoglycolic acid**	Chemical	3.15	0.0083
**2-hydroxyglutarate**	Fatty Acid, Dicarboxylate	-2.79	0.0087
**2-hydroxyphenylacetate**	Phenylalanine Metabolism	2.81	0.0100
**oleoylcarnitine (C18:1)**	Fatty Acid Metabolism (Acyl Carnitine, Monounsaturated)	-2.88	0.0112
**2R,3R-dihydroxybutyrate**	Fatty Acid, Dihydroxy	2.86	0.0122
**argininate**	Urea cycle; Arginine and Proline Metabolism	2.96	0.0134
**N2-acetyl,N6-methyllysine**	Lysine Metabolism	-2.47	0.0140
**choline**	Phospholipid Metabolism	-2.85	0.0143
**octanoylcarnitine (C8)**	Fatty Acid Metabolism (Acyl Carnitine, Medium Chain)	-2.97	0.0149
**androstenediol (3beta,17beta) monosulfate (2)**	Androgenic Steroids	-2.50	0.0166
**nicotinamide**	Nicotinate and Nicotinamide Metabolism	-2.67	0.0170
**4-ethylcatechol sulfate**	Benzoate Metabolism	3.18	0.0172
**myristoleoylcarnitine/physeteroylcarnitine (C14:1)**	Fatty Acid Metabolism (Acyl Carnitine, Monounsaturated)	-2.84	0.0176
**tetradecadienoate (14:2)***	Long Chain Polyunsaturated Fatty Acid (n3 and n6)	-2.94	0.0186
**palmitoylcarnitine (C16)**	Fatty Acid Metabolism (Acyl Carnitine, Long Chain Saturated)	-2.73	0.0188
**3-hydroxyoleoylcarnitine**	Fatty Acid Metabolism (Acyl Carnitine, Hydroxy)	-2.64	0.0192
**cholate**	Primary Bile Acid Metabolism	2.80	0.0206

**2-acetamidophenol sulfate**	Food Component/Plant	2.86	0.0207
**palmitoleoylcarnitine (C16:1)**	Fatty Acid Metabolism (Acyl Carnitine, Monounsaturated)	-2.77	0.0217
**1-linolenoylglycerol (18:3)**	Monoacylglycerol	2.85	0.0222
**ethylparaben sulfate**	Chemical	-2.53	0.0232
**4-guanidinobutanoate**	Guanidino and Acetamido Metabolism	2.84	0.0235
**indolepropionate**	Tryptophan Metabolism	2.50	0.0236
**5-oxoproline**	Glutathione Metabolism	-2.31	0.0242
**10-undecenoate (11:1n1)**	Medium Chain Fatty Acid	-2.81	0.0243
**cis-4-decenoylcarnitine (C10:1)**	Fatty Acid Metabolism (Acyl Carnitine, Monounsaturated)	-2.59	0.0245
**tetradecadienedioate (C14:2-DC)**	Fatty Acid, Dicarboxylate	-2.62	0.0256
**alpha-hydroxyisovalerate**	Leucine, Isoleucine and Valine Metabolism	-2.34	0.0269
**hexanoylglutamine**	Fatty Acid Metabolism (Acyl Glutamine)	-2.65	0.0271
**glucuronate**	Aminosugar Metabolism	2.56	0.0272
**5alpha-androstan-3alpha,17beta-diol monosulfate (2)**	Androgenic Steroids	-2.23	0.0317
**4-methoxyphenol sulfate**	Tyrosine Metabolism	2.67	0.0319
**methionine sulfoxide**	Methionine, Cysteine, SAM and Taurine Metabolism	2.48	0.0323
**decanoylcarnitine (C10)**	Fatty Acid Metabolism (Acyl Carnitine, Medium Chain)	-2.58	0.0324
**arachidonoylcarnitine (C20:4)**	Fatty Acid Metabolism (Acyl Carnitine, Polyunsaturated)	-2.26	0.0333
**myristoylcarnitine (C14)**	Fatty Acid Metabolism (Acyl Carnitine, Long Chain Saturated)	-2.66	0.0335
**linoleoylcarnitine (C18:2)**	Fatty Acid Metabolism (Acyl Carnitine, Polyunsaturated)	-2.35	0.0341
**1-docosahexaenoylglycerol (22:6)**	Monoacylglycerol	2.41	0.0345
**taurine**	Methionine, Cysteine, SAM and Taurine Metabolism	-2.56	0.0371
**aspartate**	Alanine and Aspartate Metabolism	-6.34	0.0377
**1-myristoylglycerol (14:0)**	Monoacylglycerol	2.65	0.0379

**galactonate**	Fructose, Mannose and Galactose Metabolism	-2.67	0.0402
**1-methylurate**	Xanthine Metabolism	2.23	0.0408
**succinate**	TCA Cycle	2.21	0.0410
**lyxonate**	Pentose Metabolism	-2.64	0.0420
**dimethyl sulfone**	Chemical	2.34	0.0424
**1-arachidoyl-2-arachidonoyl-GPC (20:0/20:4)**	Phosphatidylcholine (PC)	-2.34	0.0425
**branched chain 14:0 dicarboxylic acid**	Fatty Acid, Dicarboxylate	2.33	0.0433
**dihomo-linoleoylcarnitine (C20:2)**	Fatty Acid Metabolism (Acyl Carnitine, Polyunsaturated)	-2.16	0.0434
**3-methoxycatechol sulfate (2)**	Benzoate Metabolism	2.28	0.0444
**biliverdin**	Hemoglobin and Porphyrin Metabolism	-2.28	0.0450
**stearoylcarnitine (C18)**	Fatty Acid Metabolism (Acyl Carnitine, Long Chain Saturated)	-2.25	0.0451
**1-stearoyl-2-arachidonoyl-GPS (18:0/20:4)**	Phosphatidylserine (PS)	-2.58	0.0452
**gentisate**	Tyrosine Metabolism	2.42	0.0454
**oleoyl ethanolamide**	Endocannabinoid	-2.46	0.0457
**3-hydroxydecanoylcarnitine**	Fatty Acid Metabolism (Acyl Carnitine, Hydroxy)	-2.35	0.0473
**ceramide (d18:1/20:0, d16:1/22:0, d20:1/18:0)**	Ceramides	2.47	0.0477
**glycocholate**	Primary Bile Acid Metabolism	-2.33	0.0481
**3-hydroxybutyroylglycine**	Fatty Acid Metabolism (Acyl Glycine)	-2.13	0.0483
**4-acetylcatechol sulfate (1)**	Food Component/Plant	2.38	0.0495
**3-ureidopropionate**	Pyrimidine Metabolism, Uracil containing	2.43	0.0497

Note. $^{*}\beta$ is the regression coefficient of intervention in the mixed-effects model adjusted for age, race, BMI and use of antidepressant.
